# Supplementary material for: HIVprotI: an integrated web based platform for prediction and design of HIV proteins inhibitors
Source: J Cheminform. 2018 Mar 9;10:12. doi: 10.1186/s13321-018-0266-y (PMC5845081; doi:10.1186/s13321-018-0266-y)
Supplement: Supplementary file 2 — Additional file 2. Source code of HIVProtI web server. [file 13321_2018_266_MOESM2_ESM.zip › HIVprotI_Source-code/hivprotI/weka-3-6-6/doc/weka/experiment/ResultProducer.html]

ResultProducer


---


|  |  |  |
| --- | --- | --- |
| **Overview** \| **Package** \| **Class** \| **Tree** \| **Deprecated** \| **Index** \| **Help** \| **Weka's home** | |  |
| **PREV CLASS**   **NEXT CLASS** | **FRAMES**    **NO FRAMES**     **All Classes** |  |
| SUMMARY: NESTED \| FIELD \| CONSTR \| METHOD | DETAIL: FIELD \| CONSTR \| METHOD |  |


---


## weka.experiment Interface ResultProducer

**All Superinterfaces:**: java.io.Serializable

**All Known Implementing Classes:**: AveragingResultProducer, CrossValidationResultProducer, DatabaseResultProducer, LearningRateResultProducer, RandomSplitResultProducer

---

``` public interface ResultProducer extends java.io.Serializable ```

This interface defines the methods required for an object
that produces results for different randomizations of a dataset.

Possible implementations of ResultProducer:   

- Random test/train splits- CrossValidation splits- LearningCurve splits (multiple results per run?)- Averaging results of other result producers

**Version:**
:   $Revision: 1.7 $

**Author:**
:   Len Trigg (trigg@cs.waikato.ac.nz)

---

| **Method Summary** | |
| --- | --- |
| `void` | `doRun(int run)`             Gets the results for a specified run number. |
| `void` | `doRunKeys(int run)`             Gets the keys for a specified run number. |
| `java.lang.String` | `getCompatibilityState()`             Gets a description of the internal settings of the result producer, sufficient for distinguishing a ResultProducer instance from another with different settings (ignoring those settings set through this interface). |
| `java.lang.String[]` | `getKeyNames()`             Gets the names of each of the key columns produced for a single run. |
| `java.lang.Object[]` | `getKeyTypes()`             Gets the data types of each of the key columns produced for a single run. |
| `java.lang.String[]` | `getResultNames()`             Gets the names of each of the result columns produced for a single run. |
| `java.lang.Object[]` | `getResultTypes()`             Gets the data types of each of the result columns produced for a single run. |
| `void` | `postProcess()`             Perform any postprocessing. |
| `void` | `preProcess()`             Prepare to generate results. |
| `void` | `setAdditionalMeasures(java.lang.String[] additionalMeasures)`             Sets a list of method names for additional measures to look for in SplitEvaluators. |
| `void` | `setInstances(Instances instances)`             Sets the dataset that results will be obtained for. |
| `void` | `setResultListener(ResultListener listener)`             Sets the object to send results of each run to. |

**Method Detail**

### setInstances

```
void setInstances(Instances instances)
```

:   Sets the dataset that results will be obtained for.

    :   **Parameters:**: `instances` - a value of type 'Instances'.

---


### setResultListener

```
void setResultListener(ResultListener listener)
```

:   Sets the object to send results of each run to.

    :   **Parameters:**: `listener` - a value of type 'ResultListener'

---


### setAdditionalMeasures

```
void setAdditionalMeasures(java.lang.String[] additionalMeasures)
```

:   Sets a list of method names for additional measures to look for
    in SplitEvaluators.

    :   **Parameters:**: `additionalMeasures` - a list of method names

---


### preProcess

```
void preProcess()
                throws java.lang.Exception
```

:   Prepare to generate results. The ResultProducer should call
    preProcess(this) on the ResultListener it is to send results to.

    :   **Throws:**: `java.lang.Exception` - if an error occurs during preprocessing.

---


### postProcess

```
void postProcess()
                 throws java.lang.Exception
```

:   Perform any postprocessing. When this method is called, it indicates
    that no more requests to generate results for the current experiment
    will be sent. The ResultProducer should call
    preProcess(this) on the ResultListener it is to send results to.

    :   **Throws:**: `java.lang.Exception` - if an error occurs

---


### doRun

```
void doRun(int run)
           throws java.lang.Exception
```

:   Gets the results for a specified run number. Different run
    numbers correspond to different randomizations of the data. Results
    produced should be sent to the current ResultListener, but only
    if the ResultListener says the result is required (it may already
    have that result). A single run may produce multiple results.

    :   **Parameters:**: `run` - the run number to generate results for. **Throws:**: `java.lang.Exception` - if a problem occurs while getting the results

---


### doRunKeys

```
void doRunKeys(int run)
               throws java.lang.Exception
```

:   Gets the keys for a specified run number. Different run
    numbers correspond to different randomizations of the data. Keys
    produced should be sent to the current ResultListener

    :   **Parameters:**: `run` - the run number to get keys for. **Throws:**: `java.lang.Exception` - if a problem occurs while getting the keys

---


### getKeyNames

```
java.lang.String[] getKeyNames()
                               throws java.lang.Exception
```

:   Gets the names of each of the key columns produced for a single run.
    The names should not contain spaces (use '\_' instead for easy
    translation.)

    :   **Returns:**: an array containing the name of each key column **Throws:**: `java.lang.Exception` - if the key names could not be determined (perhaps because of a problem from a nested sub-resultproducer)

---


### getKeyTypes

```
java.lang.Object[] getKeyTypes()
                               throws java.lang.Exception
```

:   Gets the data types of each of the key columns produced for a single run.

    :   **Returns:**: an array containing objects of the type of each key column. The objects should be Strings, or Doubles. **Throws:**: `java.lang.Exception` - if the key types could not be determined (perhaps because of a problem from a nested sub-resultproducer)

---


### getResultNames

```
java.lang.String[] getResultNames()
                                  throws java.lang.Exception
```

:   Gets the names of each of the result columns produced for a single run.
    The names should not contain spaces (use '\_' instead for easy
    translation.)

    :   **Returns:**: an array containing the name of each result column **Throws:**: `java.lang.Exception` - if the result names could not be determined (perhaps because of a problem from a nested sub-resultproducer)

---


### getResultTypes

```
java.lang.Object[] getResultTypes()
                                  throws java.lang.Exception
```

:   Gets the data types of each of the result columns produced for a
    single run.

    :   **Returns:**: an array containing objects of the type of each result column. The objects should be Strings, or Doubles. **Throws:**: `java.lang.Exception` - if the result types could not be determined (perhaps because of a problem from a nested sub-resultproducer)

---


### getCompatibilityState

```
java.lang.String getCompatibilityState()
```

:   Gets a description of the internal settings of the result
    producer, sufficient for distinguishing a ResultProducer
    instance from another with different settings (ignoring
    those settings set through this interface). For example,
    a cross-validation ResultProducer may have a setting for the
    number of folds. For a given state, the results produced should
    be compatible. Typically if a ResultProducer is an OptionHandler,
    this string will represent those command line arguments required
    to set the ResultProducer to that state.

    :   **Returns:**: the description of the ResultProducer state, or null if no state is defined


---


|  |  |  |
| --- | --- | --- |
| **Overview** \| **Package** \| **Class** \| **Tree** \| **Deprecated** \| **Index** \| **Help** \| **Weka's home** | |  |
| **PREV CLASS**   **NEXT CLASS** | **FRAMES**    **NO FRAMES**     **All Classes** |  |
| SUMMARY: NESTED \| FIELD \| CONSTR \| METHOD | DETAIL: FIELD \| CONSTR \| METHOD |  |


---
